# Optimizing Care for Trauma Patients with Obesity

**DOI:** 10.7759/cureus.3021

**Published:** 2018-07-22

**Authors:** Sanjiv Gray, Beatrice Dieudonne

**Affiliations:** 1 Surgery, University of Central Florida, Orlando, USA; 2 Surgery, Harlem Hospital Center, New York, USA

**Keywords:** bariatrics, obesity, trauma, hypoventilation, intubation, critical care

## Abstract

Obesity is a growing epidemic that has been contributing to the increasing cost of healthcare. Its prevalence is now approximately 37%. Morbid obesity is associated with increased morbidity and mortality in trauma patients. An increased recognition of obesity as a chronic disease and a better understanding of its pathophysiology can allow for proper preparation and accommodative measures to improve resuscitation and subsequent care, thereby improving trauma outcomes. The aim of this review is to provide an overview of the scope of the problem. This review also provides evidence-based recommendations for the optimal resuscitation sequence for obese patients.

## Introduction and background

The increasing prevalence of obesity in the United States mirrors the growth and expansion of trauma systems throughout the country. Therefore, more patients with obesity are likely to be triaged to trauma centers. This poses a unique challenge to healthcare providers in their efforts to provide effective and efficient care to trauma patients with obesity. The presence of obesity affects each stage of the trauma system from prehospital to the emergency room and further to inpatient care. Adiposity affects each of the phases of resuscitation such as airway management, respiratory mechanics, monitoring and support, circulatory monitoring, and optimization. An appreciation of the limitations imposed by fat mass disease will enable safer mobilization and rehabilitation while limiting morbidity. This article aims to give an overview of the scope of the problem and provide evidence-based recommendations for the optimal resuscitation sequence for patients with obesity.

## Review

Obesity is an epidemic, now affecting approximately 37% of the population of the United States. The Obesity Medicine Association (OBA) has defined obesity as “a chronic, relapsing, multi-factorial, neurobehavioral disease, wherein an increase in body fat promotes adipose tissue dysfunction and abnormal fat mass physical forces, resulting in adverse metabolic, biomechanical, and psychosocial health consequences.” Obesity can have a negative effect in trauma patients, and trauma can have a negative effect on obesity. Obesity can also be defined as a body mass index (BMI) > 30 kg/m^2^.

In 2008, the World Health Organization (WHO) estimated that 1.4 billion adults worldwide were overweight, and more than 500 million were obese. The prevalence of obesity has nearly doubled between 1980 and 2008. Moreover, 2.8 million people are estimated to die annually due to the consequences of being overweight or obese, and obesity-related healthcare costs are estimated to be $80 billion annually. Obese patients also have a 25% increased risk of workplace injury [1]. The odds of sustaining an injury were found to be 15% to 48% higher in overweight and obese individuals than in normal-weight patients, and hospital mortality rates following trauma were reported to be 7.1 times higher in obese than in non-obese patients. However, the medical literature presents conflicting results on the impact of obesity on trauma outcomes. Early studies have found that obesity was not a risk factor, but this may have been due to the lack of a standard definition and a failure to list obesity as a diagnosis, as obesity was only recently recognized as a disease. Newer studies have shown that obesity was strongly associated with the severity of the injury, hospital length of stay (LOS), intensive care unit (ICU) admission, the pattern of injury, the rate of complications, and mortality [2-3]. Obesity was found to be associated with increased mortality and pulmonary complications, as well as with increased risks of multiorgan failure and mortality after severe blunt trauma [4-5]. Obese patients in the National Trauma Data Bank (NTDB) had a higher mortality rate after blunt trauma, suggesting that injury prevention efforts focus on obese individuals [6]. Obese children had a significantly longer hospital LOS and use of ventilators, as well as increased rates of pneumonia, deep venous thrombosis (DVT), pulmonary embolism, and mortality [7]. Rates of DVT and decubitus ulcers were higher in obese children, whereas rates of intracranial and intraabdominal injuries were lower [8]. BMI was found to be independently associated with increased fat embolism in children after trauma [9], and high BMI in children was associated with higher rates of rib and pelvic fractures and higher injury severity scores (ISS) after trauma [10].

Obese patients experience higher rates of complications and mortality after a traumatic brain injury, although this may be associated with age, lower blood pressure at admission, and increased rates of chest injury, rather than directly resulting from their obese state [11]. Obese patients may also have diagnosed or undiagnosed physiologic abnormalities, leading to compromises in cardiovascular, pulmonary, and/or metabolic physiology. These abnormalities may include dyslipidemias, hypertension (HTN), insulin resistance, and their consequences, such as coronary artery disease, cerebral vascular accident, non-alcoholic steatohepatitis, cancer, arthritis, and sleep apnea.

Comorbidities can increase morbidity and mortality, either independently or in association with other conditions. Patients with obesity have been observed to have particular patterns of injury. For example, obese patients have had fewer head injuries, but more chest and lower extremity injuries [12], and a study involving patients in head-on motor vehicle collisions (MVC) showed that obesity was associated with higher risks of lower and upper extremity injuries, as well as spinal injuries [13].

Obesity is associated with increased anterior abdominal fat content, which may protect against abdominal blunt and penetrating injuries, leading to lower rates of liver and head injuries [13]. Due to this “cushioned effect", increased BMI protected patients against abdominal stab wounds and reduced the need for surgery [14]. Obese patients injured by falls and MVCs were also found to sustain lower rates of head injuries, indicating that obesity was a protective factor in these patients [15].

Higher BMI in patients aged >45 years was found to be associated with higher rates of injuries of the upper torso and proximal upper extremities [16]. Evaluation of the “cushion effect" by comparing outcomes after MVCs in lean, overweight, and obese patients showed that overweight patients were protected from dying during MVCs, whereas obese had higher mortality rates than lean patients [17]. 

Prehospital care for patients with obesity provided challenges for emergency medical services, as obese patients required longer extraction times, increasing their risk of crush injury. These patients tend to have shorter necks and require larger collar sizes. The presence of obstructive sleep apnea (OSA) can make it difficult for obese patients to lie flat and can increase the risks of airway obstruction and aspiration. Conventional ambulance stretchers are limited by their maximum rated loads and widths, typically 300 kg and 58 cm, respectively. These transport and positioning difficulties lead to delays and increase the risk of injury to staff. Health and safety regulations require additional workforce and assistive equipment to transfer obese patients onto stretchers safely. This obstacle itself can increase extrication and transport times, delaying care of severely injured patients. Sandbags and towel rolls can be utilized to properly stabilize the cervical and thoracic spine. Other examples of useful equipment include heavy stokes stretchers and scoop baskets. Healthcare workers involved in the transportation of bariatric patients are at increased risk of back injuries. In the United Kingdom, these workers are being trained with bariquins, which mimic bariatric patients.

Initial resuscitation

The evaluation and management of bariatric patients following trauma should follow advanced trauma life-saving precepts, with attention paid to the airway, breathing, circulation, disability, and full exposure. Obesity and OSA are considered risk factors for a difficult airway, as airway obstruction can occur when these patients are lying flat. Obesity and OSA are also risk factors for difficult intubation grades and present difficulties in performing bag-mask ventilation. Many predictive scores are available to identify patients with a difficult airway. These include an elevated Mallampati score, limited mouth opening, reduced cervical mobility, presence of an OSA syndrome, coma, and severe hypoxemia (i.e., the MACOCHA score). The risk factors included in the MACOCHA score include age > 55 years, BMI > 26 kg/m^2^, snoring, beard, and lack of teeth, all of which are independent risk factors for difficult mask ventilation [18-19]. Mallampati score and thyromental distance have the highest positive predictive value and sensitivity in predicting difficult intubations. Cricothyroidotomy may prove difficult due to a large neck, a deep position of the trachea, distorted anatomy secondary to trauma, and inability to use the standard-length tracheostomy tubes. Recommendations to achieve more successful endotracheal intubation include starting with an airway examination, using a short-handled laryngoscope, and having a laryngeal mask airway available as an adjunct. A difficult airway cart containing a fiberoptic bronchoscope and video laryngoscope should be available, and nasal intubation should be avoided due to mucosal engorgement [20]. Preparation for intubation should include preoxygenation to reduce the non‐hypoxic apnea time, as desaturation during intubation occurs at less than one minute in severely obese individuals and the end‐expiratory volume is reduced by 69% after induction of anesthesia in the supine position. The main cause of this rapid desaturation is the decrease in functional reserve capacity (FRC) [21]. Using a positive end-expiratory pressure (PEEP) of 10 cm H_2_O during pre‐oxygenation reduces atelectasis surface oxygenation and increases the time of apnea without hypoxemia by an average of one minute. A high-flow nasal cannula can be used for pre‐oxygenation of obese patients, even during apneic oxygenation, allowing for oxygen delivery during the apnea period. A 30-degree reverse Trendelenburg position or a 25-degree head-up position has each been shown to improve preoxygenation PaO_2 _and safe apnea time in morbidly obese patients. The patient should be positioned so that the external auditory meatus and sternal notch line up in the horizontal axis, which is optimal for intubation.

Blankets under the shoulders are recommended in patients cleared from spinal injuries as it allows the breast tissue to fall away from the chin and neck areas. For intubated patients, it is important to remember that the Acute Respiratory Distress Syndrome Network (ARDSnet) protocol is based on height, not weight. Ventilator calculations of tidal volume should be based on ideal, not actual, body weight in order to minimize barotrauma, volutrauma, chronic hypoxia, and hypercapnia. However, while intubated, simple measures such as reverse Trendelenburg positioning and adequate PEEP may improve pulmonary function. When assessing ventilation of patients with morbid obesity, clinicians should note that abdominal pressure is increased because of increased intraabdominal adipose tissue hypertrophy. The increased abdominal pressures also pushes up on the diaphragm, reducing chest capacity. This leads to decreased pulmonary and thoracic compliance and reduced FRC, and requires increased work to breathe when compared with non‐obese patients. At rest, the oxygen consumption is 1.5 times higher in obese than in non‐obese patients, with spontaneous breathing rates of 15 to 21 breaths per minute in morbidly obese patients and 10 to 12 breaths per minute in non‐obese patients [21]. In addition, hypoventilation or obstructive sleep apnea with long‐lasting apnea and hypopnea induces a secondary depression of respiratory drive with daytime hypercapnia, leading to obesity hypoventilation syndrome, defined as a combination of obesity (BMI ≥ 30 kg/m^2^), daytime hypercapnia (PaCO_2 _> 45 mm Hg), and disordered breathing during sleep [22-23]. These prohibitory factors impair ventilation. During the postintubation period, protective low tidal volume ventilation should be performed. The IMPROVE study [24] showed that protective ventilation reduced the global rate of complications from 27.5% to 10.5% and the hospital LOS by two days. PEEP should be maintained at > 5 cm H2O with cautious monitoring of hemodynamic status. Recruitment maneuvers can be employed, keeping in mind the baby lung concept that a bigger chest wall does not mean bigger lungs. The respiratory rate should be approximately 15 to 21 breaths per minute [24].

Intubation may be avoided in some patients requiring respiratory support. Continuous positive airway pressure (CPAP) may be used for longer periods in hypercapnic obese patients to reduce hypercapnia to < 50 mmHg. High-flow nasal cannula oxygen or postoperative CPAP can be extended to all obese patients, even those without obstructive apnea syndrome, especially if they are on opiates. This should be accompanied by end-tidal carbon dioxide monitoring. Patients in respiratory distress with suspected pneumothorax should be decompressed in the anterior axillary line of the fifth intercostal space, as this has been shown to be more beneficial than decompression at the midclavicular line of the second intercostal space [25]. Decompression should be followed by tube thoracostomy. Clinicians resuscitating morbidly obese patients should use appropriately sized sphygmomanometer cuffs, as these patients may have a greater need for invasive blood pressure monitoring. Patients with obesity have increased blood volume and hence higher cardiac output. This continuous myocardial stress causes ventricular hypertrophy and decreases compliance. This results in atrial dilatation, which increases the risk of arrhythmias and sudden death. Therefore, a screening electrocardiography should be performed and the patient placed on telemetry. In addition, obesity was found to be directly related to a higher rate of massive transfusion [26].

During exposure, there may be difficulty performing a log roll. Additional staff will be needed to ensure patient and staff safety. Patients may have chronic back pain and are at high risk of nerve entrapment and palsy. Secondary diagnostic imaging may be necessary but has its own technical challenges due to beam penetration and image quality, reducing sensitivity for injuries. Therefore, one should have a low threshold for obtaining computed tomography (CTs). However, the patient's size and weight may preclude computed tomography and magnetic resonance imaging (MRI), necessitating a transfer to the appropriate facility or the use of other diagnostic modalities. 

Critical care

Obese patients are often admitted to ICUs after trauma due to polytrauma, hypoxemia, and the need for greater care. The number of days in the ICU and mechanical ventilation were found to be significantly greater in obese than in non-obese patients, and obese patients have a higher rate of complications, such as acute kidney injury and infections. A study of trauma patients who needed temporary abdominal closure (TAC) found that the LOS in the ICU was significantly greater in morbidly obese (BMI >40 kg/m^2^) than in normal weight (BMI <25 kg/m^2^) patients, and that mechanical ventilation was longer in patients with BMI > 40 kg/m^2 ^than in patients with BMI < 18.5 kg/m^2^ [27]. There was no difference in survival or type of abdominal closure among these groups, indicating that TAC was safe in trauma patients with BMI >30 kg/m^2^ [27]. Obese trauma patients admitted to the ICU had a higher rate of adult respiratory distress syndrome (ARDS), required two additional days of mechanical ventilation, and more often failed attempted extubation than non-obese patients. Morbidly obese patients with traumatic injury had a higher multiorgan failure (MOF) rate [28-29], as well as four-fold higher and longer hospital stays [29].

Angiotensin-converting enzyme inhibitors and angiotensin receptor blockers may improve outcomes following trauma in severely injured obese patients by reducing angiotensin II levels [30]. A greater understanding of the pathophysiology of the obesity may allow more direct therapy to improve survival in the injured morbidly obese patients. Hormones secreted by adipocytes, such as leptin and interleukin-10, have immunomodulatory properties, with leptin regulating T-lymphocytes and interferon production. Clinical studies in humans have reported higher leptin concentrations in survivors than in non-survivors of severe sepsis and septic shock. Leptin is thought to curb inflammatory responses and improve host survival, leading to improved ICU and sepsis management [30]. The obesity paradox was reported in a study of 108,927 patients with acute decompensated heart failure [31]. The in-hospital mortality rates in underweight, normal weight, overweight, and obese patients were 6.3%, 4.6%, 3.4%, and 2.4%, respectively [31].

Nutritional requirements for obese patients should follow recently formulated Aspen guidelines. Nutrition should be assessed and a nutrition support plan developed within 48 hours of ICU admission, with energy requirements based on the Penn State University 2010 predictive equation, or the modified Penn State equation if the patient is over age 60 years [32]. The clinical outcomes are at least equivalent in patients supported with high-protein and hypocaloric feeding and those supported with high-protein and eucaloric feeding. A trial of hypocaloric, high-protein feeding is suggested in patients who do not have severe renal or hepatic dysfunction [33]. Hypocaloric feeding may start at 50% to 70% of estimated energy needs or < 14 kcal/kg actual weight. High protein feeding may be started at 1.2 g/kg actual weight or 2 to 2.5 g/kg of ideal body weight, with protein intake adjusted according to the results of nitrogen balance assessments. Hyperinsulinemia prevents the mobilization of fats, so obese patients use more proteins and carbohydrates.

Obese patients lose proteins at an accelerated rate during critical illnesses as they tend to use muscle as their source of fuel [34]. Because post-bariatric surgery patients are at increased risk of nutrient deficiency, hospitalized patients with a history of bariatric surgery should be evaluated for depletion of iron, copper, zinc, selenium, thiamine, folate, and vitamins B12 and D, with repletion of any deficiencies. Caution should be exercised when inserting feeding tubes into patients after bariatric surgery to prevent injury. Subcutaneous ports should be accessed in critically ill patients with adjustable gastric bands, particularly those who have given an unreliable abdominal examination, followed by a decompression of the adjustable band.

Early mobility has been shown to improve mortality and morbidity in the ICU. Using functional independence measurement (FIM) to measure the delay in recovery following trauma, it was found that every 1 kg/m^2^ increase in BMI correlated with a 4% decrease in FIM. Early mobility may be facilitated by assessing pre-injury mobility and use of assisting devices, by having the appropriate equipment such as bariatric beds and hoists available, and by using safe moving techniques. Because patients with a BMI ≥25 kg/m^2 ^were found to be at significantly higher risk of developing rhabdomyolysis, creatinine kinase levels and renal function should be monitored. Obesity is an established risk factor for chronic kidney disease, as intraabdominal hypertension (HTN) causes poor perfusion of the kidneys [34].

Obesity has implications for operative management because of the longer times required to perform certain aspects of care, the need for customized equipment, compromised imaging, difficulties with positioning and postoperative care, and wound complications. Wound healing and deep infection are significant concerns due to the need for greater exposure and compromised blood supply to the adipose tissue. Obese individuals have a higher incidence of vitamin D deficiency, which may have an adverse effect on wound healing. Obese patients undergoing laparotomy had increased LOS in the ICU as well as increased rates of respiratory and renal failure, bacteremia, and abdominal wound dehiscence [33].

Decubitus ulcer development and wound complications are four to eight-fold and 2.5 to four-fold higher, respectively, in severely obese patients [35], with these patients also having a higher risk for venous thromboembolism (VTE). A prospective study evaluating clotting factor concentrations in obese patients following injury showed that platelet counts and factor IX levels were elevated and D-dimer levels reduced [36]. Moreover, each 5 kg/m^2 ^increase in BMI was associated with an 85% increased likelihood of thromboembolic complications [36]. The pro-thrombotic state was associated with a slower recovery time and longer immobility. The standard of care categorizes obese patients after trauma as being at high risk for VTE events, requiring preventive measures and use of unfractionated heparin or low molecular weight heparin (LMWH) and/or pneumatic compression, as indicated. The distribution of LMWH is weight based; therefore, the efficacy of standard doses may be reduced because obesity affects plasma drug distribution and pharmacokinetics. The ITOHENOX study [37] showed that, compared with a standard enoxaparin regimen, thromboprophylaxis with enoxaparin 60 mg in obese medical inpatients provided better control of anti-Xa activity, without increasing bleeding complication. In the PREVENT trial [38], dalteparin lost its thromboprophylactic benefit in patients with BMI >35 kg/m^2^. Weight-based dosing of enoxaparin (0.5 mg/kg once daily) for VTE prophylaxis in extremely obese medically ill patients resulted in peak anti-Xa levels within or near the recommended range for thromboprophylaxis, with no evidence of excess anti-Xa activity [39]. Consultation with a pharmacist is important for dosing and monitoring of anticoagulants, especially at extremes of weight.

Finally, there is a risk of discrimination and negative attitudes regarding patients with obesity. Education of hospital and clinical staff members is key to mitigate this risk. Providers must be aware that patients are not their diseases and that obesity, like all chronic diseases, should be acknowledged as such, accommodations made, and active management performed.

## Conclusions

Obesity is a chronic disease affecting a large portion of the United States population. Recognizing this disease and modifying treatments will help avoid complications and improve outcomes in obese patients with traumatic injuries.
